# Age-Period-Cohort Analysis of the Sex Differences in Cancer Mortality Rates in Japan from 1995 to 2015

**DOI:** 10.31557/APJCP.2020.21.6.1759

**Published:** 2020-06

**Authors:** Tasuku Okui

**Affiliations:** *Medical Information Center, Kyusyu University Hospital, Fukuoka city, Japan. *

**Keywords:** Cohort effects, vital statistics, mortality, cancer

## Abstract

**Background:**

The current study aimed to analyze the sex differences in cancer mortality rates in Japan via an age-period-cohort (APC) analysis.

**Methods:**

We used data about cancer mortality rates from 1995 to 2015 in Japan based on the Vital Statistics survey. In addition to the data about mortality from all sites of cancer, we specifically used data about stomach, lung, colorectal, and liver cancers. A Bayesian APC analysis was performed to identify changes in mortality rates based on three effects, which were as follows: age, period, and cohort. Then, we finally calculated the mortality rate ratios for each effect between men and women.

**Results:**

The sex differences in age-adjusted mortality rates for all-sites cancer, lung cancer, and liver cancer were decreasing from 1995 to 2015, and the mortality rate ratios in terms of sex decreased from 2.17 in 1995 to 1.93 in 2015. Based on the results of the APC analyses, only minimal changes were observed in the mortality rate ratios for all types of cancer between men and women during the analyzed periods. The cohort effects began to decrease from the early 20^th^ century in all types of cancer in both men and women, and the mortality rate ratios for all types of cancer between men and women began to increase in the cohorts born from 1926 to 1935. For all-sites cancer, the ratio increased from 0.49 (0.44, 0.57) in the cohort born from 1926 to 1930 to 0.81 (0.60, 1.03) in the cohort born from 1971 to 1975.

**Conclusion:**

The sex differences in cancer mortality rates were decreasing in the more recent born generations in Japan. If this trend continues, there will be a minimal difference in the morality rates in the following generations.

## Introduction

Japan is one of the countries worldwide with the highest average life expectancy, and its life expectancy is continuously increasing. Although the average life expectancies among men and women are increasing, a significant difference was observed in the values according to sex (Uchida et al., 2018). Cancer is the main cause of death among individuals in Japan, and the mortality rate is significantly higher in men than in women (Utada et al., 2012; Katanoda et al., 2015). Although previous studies have confirmed the trends in the mortality rate or age-adjusted mortality rate of each type of cancer according to sex (Qiu et al., 2009), sex differences in terms of age, period, or cohort effects on cancer mortality have not been investigated yet. The trends in the mortality rates of different types of cancer are often similar among men and women (Ito et al., 2011). However, the degree of decrease in period or cohort effects on mortality is considered to be different according to sex. 

To investigate the trend of a disease, age-period-cohort analysis (APC) is often performed (Smith and Wakefield, 2016). The APC analysis is specifically used in the public health domain to analyze trends in the incidence or mortality rate of a disease, and it can identify changes in statistics based on age, period, and cohort effects. This method has been used to investigate mortality from representative cancer subtypes not only in Japan (Takahashi et al., 2001; Ito et al., 2011; Tanaka et al., 2012; Shin et al., 2013; Wang et al., 2015) but also in other countries (Liaw et al., 2005; Shin et al., 2013; Ho et al., 2015). However, the periods analyzed are different for each type of cancer, and APC analyses using data obtained recently have not been conducted in Japan. In addition, although age, period, and cohort effects are usually assessed according to sex, the mortality rate ratios or differences in sex for each effect are not usually evaluated, and whether the rate ratios are increasing or not is not fully elucidated.

In this study, we analyzed the trends in the mortality rate of cancer via an APC analysis of data collected from the Vital Statistics in Japan. Moreover, the morality rate ratios for each effect were determined.

## Materials and Methods

We used data from the Vital Statistics in Japan (Ministry of Health, Labour and Welfare of Japan, 2020). We used the data for the mortality from the five Vital Statistics for every 5-year period from 1995 to 2015. We used the mortality of cancer (all sites) and those of stomach, lung, colorectal, and liver in the analysis. These cancer types were selected because the mortalities were relatively large. The ICD10 code for cancer of any sites is C00-97. In addition, the codes for individual cancer types are as follows: stomach, C16; lung, C33–34; colorectal, C18–20; and liver, C22. The population statistics data of sex and age group were obtained from the Vital Statistics (Ministry of Health, Labour and Welfare of Japan, 2020).

We used the Bayesian APC model for the analysis. In order to calculate risk ratios of sex and their credible intervals for each effects, we used the model that simultaneously modeling the of both sex. Let y_ijr_ as the mortality for the age group i (1,…,I) in year j (1,…,J) of sex r (1,2). In the model, y_ijr_ are assumed to follow the following Poisson distribution whose mean are λ_ijr_. 

y_ijr_~Poisson (λ_ijr_),

$\log\left(\lambda_{\mathrm{ijr}}\right)=\delta_{r}+\alpha_{\mathrm{ir}}+\beta_{\mathrm{ir}}+\gamma_{\mathrm{jr}}+Z_{\mathrm{ijr}}+\log(n_{\mathrm{ijr}})$

where δ_r_ are the intercepts by sex, α_ir_ are the effects of age groups by sex, β_jr_ are period effects by sex, γ_kr _(k=1,.., K) are cohort effects by sex, z_ijr_ are random effects that are defined for each sex, year and age group, and n_ijr _are the corresponding population. For the identifiability of the parameters, the restriction that $\sum_{i=1}^{1}a_{\mathrm{ir}}=\sum_{j=1}^{J}\beta_{\mathrm{jr}}=\sum_{k=1}^{K}\gamma_{\mathrm{kr}}=0$ is put.

z_ijr_ are assumed to be generated from normal distribution whose mean is zero. For the priors of each effect, random walk of the first order was used to identify the parameters.

Age groups were defined in 5-year units from 40–44 years to 90–94 years in the data. Regarding the birth cohort, the cohort who were born from 1901 to 1905 is the first cohort, and 5-year shift, the cohort born from 1971 to 1975 is the last cohort in the analysis. To estimate the parameters, we used the Hamiltonian Monte Carlo method (Stan Development Team, 2020). All statistical analyses were conducted using R 3.5.1 software (R Core Team, 2020). 

## Results

Tables 1 and 2 show the mortality rates for each type of cancer according to periods and age groups. For all types of cancer, the mortality rates in all age groups, except for the 90–94-year-old age group, were decreasing from 1995 to 2015, and the trends were similar between men and women.

Table 3 depicts the age-adjusted mortality rates per 1,000 persons and the mortality rate ratios between men and women. The age-adjusted rate of each combination of sex and period was calculated using the population of men in 1995 as the standard population. The mortality rates of all types of cancer were lower in women than in men. Moreover, the mortality rates of all types of cancer were decreasing among men and women during the periods analyzed. The rate ratios of all-sites cancer, lung cancer, and liver cancer were decreasing during the periods. By contrast, those of stomach and colorectal cancer were increasing.

**Figure 1:**
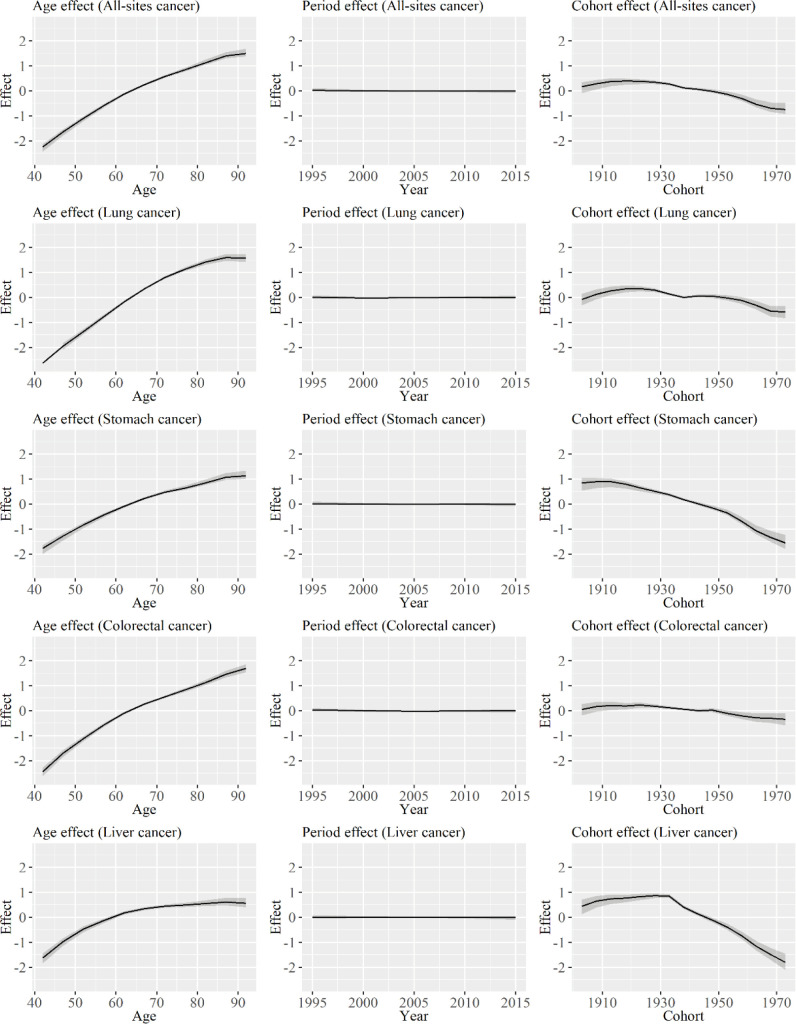
Age, Period, and Cohort Effect Estimates According to Specific Types of Cancer among Men. The graph shows the age, period, and cohort effects on all-sites cancer, lung cancer, stomach cancer, colorectal cancer, and liver cancer for men. Solid lines signify estimates of each effect, and the shadings show the credible intervals of each effect estimates. Estimates of each point are connected for visualization

Figure 1 shows age, period, and cohort effect estimates according to specific types of cancer among men. The age effects increased with increasing age in all types of cancer. However, the degree of increase was relatively low in liver cancer. Minimal changes were observed in the period effects, and those of all-sites and liver cancer had decreasing trends. The cohort effects of all-sites cancer increase from the cohort born from 1901 to 1905 to the cohort born from 1916 to 1920, and it decreased thereafter. Although subtle differences were observed in the turning points of changes according to cancer types, the cohort effects for all types of cancer had similar changes. The degree of decrease in the cohort effects for stomach cancer was the highest. However, the degree of decrease for lung and colorectal cancer was relatively lower than that for other types of cancer.

**Figure 2:**
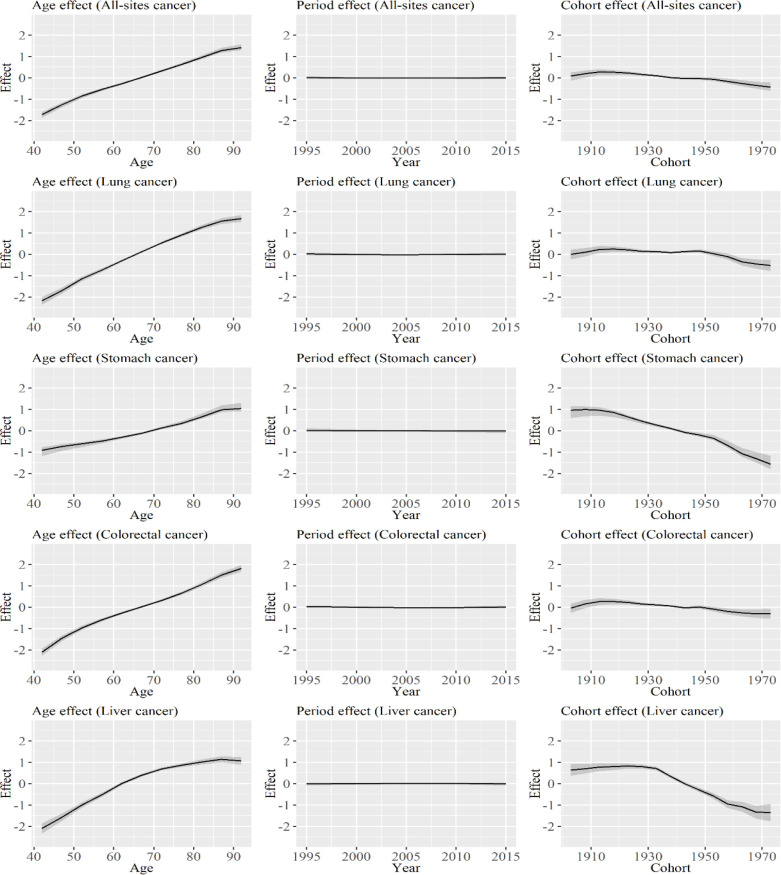
Age, Period, and Cohort Effect Estimates According to Specific Types of Cancer among Women. The graph shows the age, period, and cohort effects on all-sites cancer, lung cancer, stomach cancer, colorectal cancer, and liver cancer for women. Solid lines signify estimates of each effect, and the shadings show the credible intervals of each effect estimates. Estimates of each point are connected for visualization

Figure 2 shows age, period, and cohort effect estimates according to specific types of cancer among women. The degree of increase in the age effects for all-sites cancer in women was lower than that in men, and a similar trend was observed for stomach cancer. By contrast, the degree of increase in the age effects for liver cancer in women was significantly higher than that in men. Minimal changes were observed in the period effects, and only that of stomach cancer was decreasing during the periods. The cohort effects for all types of cancer began to decrease from the early 20th century, and no significant differences were observed in the turning points of changes among cancer types. 

**Figure 3:**
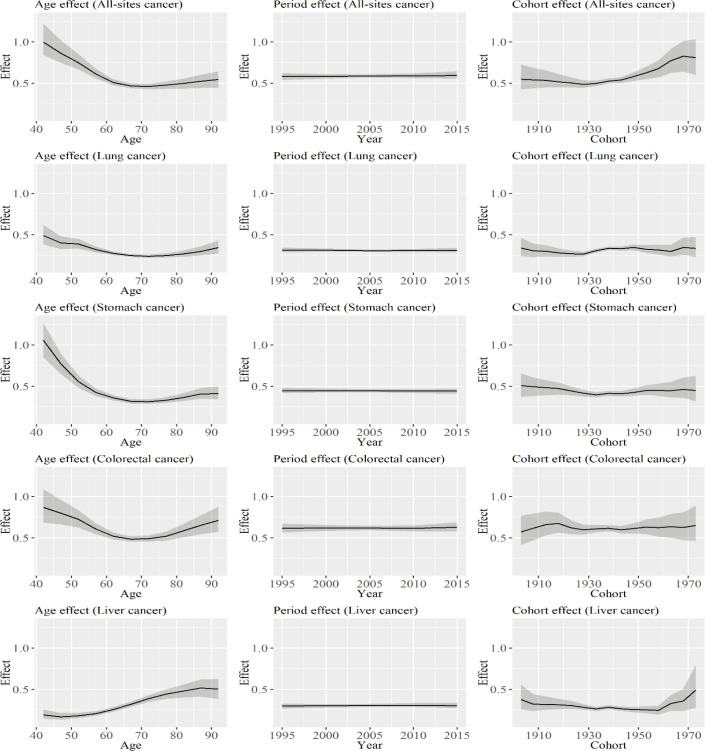
Rate Ratios for Mortality from Specific Types of Cancer between Men and Women Based on the APC Analysis. The graph shows the rate ratios for mortality about age, period, and cohort effects. Solid lines signify estimates of each ratio, and the shadings show the credible intervals of each ratio. Estimates of each point are connected for visualization

Figure 3 shows rate ratios for mortality from specific types of cancer between men and women based on the APC analysis. The rate ratio at the age of 40–45 years for all-sites cancer was 1. Then, the ratio decreased until the age of 70–75 years and it increased thereafter. Therefore, the difference in mortality rates based on age for all sites-cancer among men and women began to increase at around 40 years. Those of lung, stomach, and colorectal cancer had similar trends. By contrast, the rate ratio for liver cancer was more likely to increase with increasing age. Minimal changes were observed in the rate ratios during the period for all types of cancer. The rate ratios for all sites cancer decreased from the cohorts born from 1901 to 1905 to the cohorts born from 1926 to 1930, and it increased thereafter. Although those of lung, stomach, and liver cancer also showed increasing trends, the degree of increase was the highest in all-sites cancer.

**Table 1:** Mortality Rate of Each Cancer Type Per 100,000 Persons among Men from 1995 to 2015

Cancer types, periods, and age groups	40–44	45–49	50–54	55–59	60–64	65–69	70–74	75–79	80–84	85–89	90–94
All-sites cancer											
1995	45.4	103.2	176.5	319.6	612.1	957.0	1378.9	1797.5	2416.9	3137.8	2695.3
2000	41.9	87.0	168.8	310.2	508.9	869.5	1255.6	1685.3	2312.4	2877.9	2710.0
2005	30.7	71.1	150.5	271.9	474.2	720.8	1160.7	1672.9	2230.8	2935.7	3365.1
2010	27.0	56.2	122.9	249.9	423.2	684.9	1002.5	1539.3	2225.9	2943.0	3525.3
2015	24.9	46.6	98.5	203.3	386.9	624.9	966.0	1347.5	2022.6	2826.9	3493.5
Lung cancer											
1995	7.2	16.3	27.8	49.2	100.8	206.9	349.2	475.6	598.6	681.1	465.3
2000	7.4	14.3	28.2	51.7	89.6	173.1	307.2	451.9	596.2	660.0	502.5
2005	5.8	13.4	26.5	52.6	97.7	147.4	270.2	445.5	601.7	706.1	664.6
2010	3.9	10.8	24.3	50.6	92.2	163.2	237.1	384.4	596.0	729.0	793.6
2015	4.2	8.8	20.0	41.7	88.2	155.4	252.1	331.7	503.4	703.7	783.3
Stomach cancer											
1995	9.4	21.3	34.8	64.4	115.7	179.8	267.2	349.8	496.1	675.6	613.3
2000	7.8	16.3	31.0	57.5	91.1	156.2	225.3	299.7	435.5	580.6	561.5
2005	4.6	11.3	26.3	45.3	77.4	124.0	189.9	264.6	366.4	523.0	645.1
2010	3.8	7.6	18.3	39.3	66.3	107.1	163.1	236.7	329.8	468.1	620.7
2015	2.9	5.9	13.2	26.5	52.6	87.4	136.9	196.1	284.1	397.9	512.3
Colorectal cancer											
1995	5.4	12.3	22.2	40.3	68.3	105.0	138.2	180.6	255.4	375.0	358.4
2000	4.3	10.6	21.3	37.5	62.4	94.9	136.8	187.2	235.2	336.4	369.8
2005	4.1	9.4	18.3	35.4	55.5	84.7	127.0	179.1	246.0	308.8	423.0
2010	4.2	8.6	16.8	31.5	59.9	82.6	118.6	161.4	230.8	345.7	448.7
2015	3.9	8.6	15.5	30.1	52.9	88.2	109.7	158.3	221.7	320.6	453.9
Liver cancer											
1995	6.0	17.2	30.2	57.2	140.3	170.1	174.9	170.3	183.5	189.2	129.2
2000	4.8	12.4	27.6	51.3	89.4	164.8	173.0	172.4	186.9	177.5	142.4
2005	3.0	8.3	22.4	38.1	65.8	102.4	175.6	188.4	189.5	182.6	180.4
2010	2.3	5.7	14.4	29.3	49.9	76.1	113.3	183.3	198.2	198.2	188.1
2015	1.4	3.8	9.3	20.0	35.6	56.0	87.9	122.9	187.2	213.5	205.8

**Table 2:** Mortality Rate of each Cancer Type Per 100,000 Persons among Women from 1995 to 2015

Cancer types, periods, and age groups	40–44	45–49	50–54	55–59	60–64	65–69	70–74	75–79	80–84	85–89	90–94
All-sites cancer											
1995	48.0	85.9	121.1	173.5	255.3	365.6	523.6	761.8	1077.9	1512.1	1344.2
2000	46.9	80.0	124.2	171.0	229.6	340.2	482.9	691.7	992.9	1362.4	1323.7
2005	41.2	69.4	118.7	165.3	220.4	305.0	456.2	648.9	954.6	1324.6	1657.5
2010	37.3	62.6	105.6	162.4	215.6	296.4	416.1	614.5	900.4	1308.5	1691.0
2015	34.1	58.4	97.8	145.9	212.3	285.5	405.1	577.1	861.9	1241.3	1660.2
Lung cancer											
1995	3.9	7.2	11.9	16.5	28.5	43.4	70.4	110.5	149.6	195.3	176.8
2000	3.8	5.9	12.8	18.2	26.9	42.8	62.6	97.6	144.6	188.7	170.6
2005	2.1	5.1	10.9	17.8	26.3	38.5	63.1	92.6	140.9	187.9	226.8
2010	2.3	4.5	8.6	15.9	28.0	42.8	61.6	90.8	134.0	190.4	230.4
2015	2.1	3.8	7.2	14.7	25.1	43.4	64.4	89.5	132.8	178.7	226.4
Stomach cancer											
1995	10.7	14.6	18.4	25.2	35.7	51.7	80.3	123.5	196.6	317.8	282.9
2000	8.0	13.5	17.1	22.3	32.5	43.8	66.1	103.6	166.2	254.7	241.2
2005	5.0	8.4	13.8	18.7	25.6	37.3	563.0	84.0	133.1	224.6	283.0
2010	3.8	5.8	10.3	16.3	22.5	30.5	46.7	68.6	111.4	182.2	267.8
2015	3.0	5.0	7.1	11.6	18.6	27.0	39.0	56.5	91.2	149.0	215.9
Colorectal cancer											
1995	4.4	9.3	16.0	24.8	34.7	47.3	69.0	101.9	155.5	242.0	226.0
2000	4.3	9.2	15.5	19.9	32.5	46.5	62.6	96.5	152.4	232.0	260.4
2005	3.7	7.3	13.3	21.4	27.2	41.2	59.8	59.8	141.1	220.2	322.8
2010	3.2	6.5	10.8	19.5	31.2	38.8	57.7	57.7	130.8	216.1	330.5
2015	3.6	7.2	12.0	19.4	27.8	41.8	54.3	54.3	126.2	205.9	333.4
Liver cancer											
1995	1.0	2.4	4.5	10.9	28.0	48.9	63.5	63.5	88.8	101.9	79.3
2000	0.7	1.6	4.2	9.1	21.4	45.3	64.5	64.5	89.9	97.6	72.7
2005	0.7	1.0	3.4	6.5	15.0	31.1	61.7	61.7	92.6	65.6	93.4
2010	0.4	1.0	2.1	5.0	11.6	22.6	42.8	42.8	89.4	107.2	100.7
2015	0.5	0.8	1.8	3.4	7.7	14.1	27.8	27.8	78.7	97.4	101.2

**Table 3:** Age-Adjusted Mortality Rates per 1,000 persons and Rate Ratios between Men and Women from 1995 to 2015

Cancer types and periods	All-sites cancer	Lung cancer	Stomach cancer	Colorectal cancer	Liver cancer
Men					
1995	5.56	1.19	1.10	0.61	0.78
2000	5.09	1.11	0.93	0.58	0.69
2005	4.71	1.07	0.78	0.54	0.57
2010	4.35	1.02	0.68	0.52	0.46
2015	3.94	0.95	0.55	0.50	0.35
Women					
1995	2.56	0.31	0.41	0.35	0.23
2000	2.38	0.29	0.35	0.33	0.21
2005	2.26	0.28	0.29	0.30	0.18
2010	2.14	0.27	0.24	0.29	0.15
2015	2.04	0.27	0.19	0.28	0.11
Rate ratios					
1995	2.17	3.84	2.68	1.74	3.39
2000	2.14	3.83	2.66	1.76	3.29
2005	2.08	3.82	2.69	1.80	3.17
2010	2.03	3.78	2.83	1.79	3.07
2015	1.93	3.52	2.89	1.79	3.18

## Discussion

As shown in Table 3, the age-adjusted mortality rate ratios for all-sites cancer, colorectal cancer, and liver cancer had decreasing trends during the periods. The number of deaths from lung cancer was the highest in all types of cancer among men (Katanoda et al., 2015), and a decrease in the mortality rate ratio for lung cancer is correlated to that of all-sites cancer. Lung cancer is correlated to smoking habit, and the proportion of smokers was higher in men than in women in Japan. The proportion of smokers was consistently decreasing in Japan during the periods analyzed (Funatogawa et al., 2013). Thus, it is considered correlated to the incidence of lung cancer. By contrast, the rate ratio for stomach cancer is increasing, and this result is attributed to the degree of decrease in mortality rate, which is higher in women than in men. The main cause for the decrease in mortality rates for stomach cancer is associated with the reduction in the prevalence of Helicobacter pylori infection (Inoue, 2007) and salt intake (Tanaka et al., 2012), and the degree of decrease in the prevalence of Helicobacter pylori or salt intake may differ according to sex.

As shown in Table 3, the mortality rate ratio for all-sites cancer was high at the age of 40–44 years, and it decreased with age. Moreover, the mortality rates of all-sites cancer for men and women began to differ at the age of 40–44 years. Although the rate ratios were minus 1 for lung, colorectal, and stomach cancer, no difference was observed in terms of the ratio according to sex at the age of 40–44 years. This result is partially attributed to mortality from breast cancer. The mortality rate of breast cancer is relatively higher than that of other types of cancer among younger-aged individuals (Saika and Sobue, 2009), and it increases the mortality rate of all-sites cancer among women. The rate ratios of all-sites cancer, lung cancer, stomach cancer, and colorectal cancer began to increase at the age of 70–74 years, and this phenomenon is attributed to the mortality rates of women, which significantly increased around these years. 

By contrast, only a minimal change was observed in the rate ratio during the periods that were analyzed. Therefore, the increase or decrease in the age-adjusted mortality rate ratios, as shown in Table 3, is primarily caused by the change in cohort effects. Based on the result of the cohort effects in Table 6, the increase in the ratios were observed in all types of cancer. In addition, the turning point for the increase was about 10 years after the period when the decrease in cohort effects was observed, as shown in Figures 1 and 2. Therefore, the degree of decrease in the cohort effects was larger in men than in women. Results showed that the mortality rate ratio of all-sites cancer increased to 0.81 (0.60, 1.03) in cohorts born from 1971 to 1975, and the sex differences in cancer mortality rates decreased in cohorts born more recently. The result might be associated with the cohort effect on breast cancer among women. Although the cohort effect on breast cancer decreased in Japan (Wang et al. 2015), the age-adjusted mortality rate of breast cancer did not decrease in Japan (Yang et al. 2010; Wang et al. 2015). Therefore, the degree of decrease for breast cancer might be lower than that of the other types of cancer. If the mortality rate ratio for the cohort continues to decrease and that for period effect remains stable, no significant differences will be observed in terms of mortality rate according to sex in the following generations.

In this study, we analyzed the sex differences in cancer mortality rates in Japan from 1995 to 2015. Results showed that sex differences in age-adjusted mortality rates for all-sites cancer, lung cancer, and liver cancer were decreasing during the periods analyzed. Based on the results of the APC analyses, there were minimal changes in the mortality rate ratios for the periods analyzed in all types of cancer between men and women. By contrast, between men and women, the mortality rate ratios began to increase in the cohorts born from 1926 to 1935 in all types of cancer. For all-sites cancer, the ratio increased from 0.49 (0.44, 0.57) in the cohort born from 1926 to 1930 to 0.81 (0.60, 1.03) in the cohort born from 1971 to 1975.
